# Radiopharmaceutical Switch Maintenance for Relapsed Ovarian Carcinoma

**DOI:** 10.3390/ph13100287

**Published:** 2020-09-30

**Authors:** Charles A. Kunos, Jacek Capala

**Affiliations:** 1 Cancer Therapy Evaluation Program, Division of Cancer Treatment and Diagnosis, National Cancer Institute, Bethesda, MD 20850, USA; 2 Radiation Research Program, Division of Cancer Treatment and Diagnosis, National Cancer Institute, Bethesda, MD 20850, USA; capalaj@mail.nih.gov

**Keywords:** switch maintenance, continuation maintenance, radiopharmaceutical, ovarian cancer, ovarian carcinoma

## Abstract

Switch maintenance, or using alternative therapeutic agents that were not administered during a prior course of cancer treatment, has emerged as an active clinical research and regulatory agency-approvable path in the National Cancer Institute (NCI) Cancer Therapy Evaluation Program (CTEP) drug-development sequence. To better inform the design of therapeutic radiopharmaceutical trials, we reviewed academic scholarship discussing the clinical use of maintenance approaches to cancer treatment. Women with advanced-stage primary platinum-refractory or platinum-resistant ovarian carcinoma and their courses of treatment provide context for our discussion. Twenty-four (10%) out of 244 trials for women with ovarian carcinoma fit our search terms for maintenance trials. Five (2%) trials studied radiopharmaceuticals as switch maintenance. In our opinion, radiopharmaceutical switch maintenance merits further testing in prospective trials for women with advanced-stage primary platinum recurrent or refractory ovarian carcinoma.

## 1. Introduction

In 2020, one percent (21,750 women) of all new cancer cases in the United States are women newly diagnosed with ovarian carcinomas [1]. These women account for two percent (13,940 women) of all cancer deaths in the United States [1]. Conventional first course treatment involves maximal safe abdominal cytoreductive surgery followed by platinum-based chemotherapy [2]. Women with primary platinum-refractory disease, meaning their ovarian carcinoma progresses less than six months after completing first-line platinum-based chemotherapy, have a median survival estimate of only about one year [3]. Thus, there is an unmet therapeutic need for safe and effective treatment for an estimated 5438 (25% [4]) newly diagnosed American women each year whose ovarian carcinoma falls into the primary platinum-refractory disease category.

**Figure 1:**
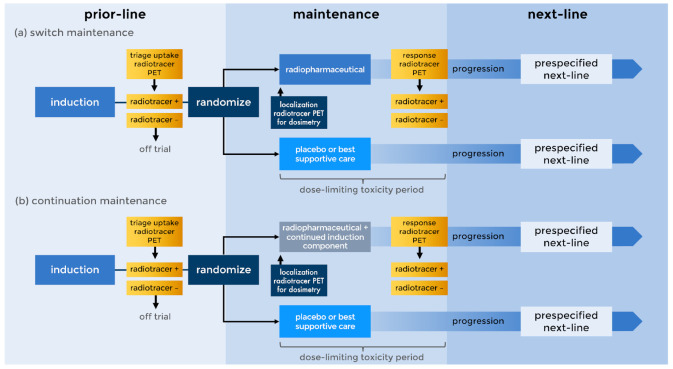
Depicted are two examples of maintenance trials. (**a**) Switch maintenance. Induction prior-line (or first-line) therapy precedes a triage scan for theranostic radiotracer uptake by positron emission tomography (PET). A subject with PET-avid disease randomizes to the investigational radiopharmaceutical or to placebo/best supportive care. A localization PET using a radiotracer can be used to determine first-cycle dosimetry. At a prespecified assessment time point, a response evaluation can be done using the same PET theranostic radiotracer. If progression occurs, then a subject starts a prespecified next-line (or second-line) therapy. (**b**) Continuation maintenance. Here, trial treatment and surveillance are the same as switch maintenance, but at least one induction component is carried forward to the maintenance treatment. Single-arm phase 0, I, or II trials for biomarker-driven studies, first-in-human or initial combinatorial safety evaluations, or efficacy in rare tumor populations might not need placebo/best supportive care arms.

A switch maintenance approach for primary platinum-refractory ovarian carcinoma has gained interest and recent regulatory approval [5,6]. Such an approach largely fits into one of two therapeutic strategies—(i) a switch maintenance strategy, whereby after conventional first-line therapy patients are switched to an alternative agent or combination of agents until disease progression, or (ii) a continuation maintenance strategy, whereby one treatment of the first-line is continued past its standard duration alone or in a new combination until disease progression [7]. For example, the PRIMA randomized double-blind trial (2016–2018) studied once daily niraparib, an inhibitor of poly(adenosine diphosphate (ADP)-ribose) polymerase (PARP), as switch maintenance after first-line platinum-based chemotherapy in 487 women with advanced-stage ovarian carcinoma [5]. Therapeutic success, as measured by prolonged median progression-free survival (22 months versus 10 months; hazard ratio for disease progression or death, 0.43; 95% confidence interval [CI], 0.31–0.59; *p* < 0.001), led to regulatory approval [5,6]. Switch maintenance has emerged as an active clinical research and regulatory agency-approvable path in the United States National Cancer Institute (NCI) Cancer Therapy Evaluation Program (CTEP) drug-development sequence. For modern radiopharmaceuticals, a maintenance strategy might more broadly squeeze between prior-line and next-line treatment, such that radiopharmaceuticals are not necessarily boxed-in by first-line and second-line therapies (Figure 1).

Early-phase trials can be very effective tools for determining whether a maintenance approach has an anticipated biological effect [8,9] or has a quick impact upon a primary endpoint [7]. To provide decisive data for the clinical benefit of a maintenance strategy, four trial design concerns must be thought about—(i) uniform exposure to a prior-line therapy, (ii) choice of next-line therapy at progression, (iii) between-arm differences in clinical follow-up, and (iv) selection of the primary endpoint, favoring overall survival or a quality of life measure [7].

As NCI CTEP continues investment in its radiopharmaceutical-agent combination portfolio, a maintenance approach for radiopharmaceutical treatment has arisen as an attractive design in our opinion for the treatment of women with primary platinum-refractory or recurrent ovarian carcinoma. Our opinion is founded upon prior early-phase radioimmunotherapy trials described here and new agent drug-development sequence opportunities in the CTEP portfolio.

## 2. Results

A search for clinical trials engaging either a switch or a continuation maintenance approach (i.e., fitting trial designs in Figure 1) for the treatment of women with ovarian carcinoma was conducted from among published academic scholarship, or, more broadly, from NCI CTEP databases listing active or completed solid tumor or hematological cancer trials. After searching academic scholarship, 244 trials were deemed eligible for inclusion in our retrospective review. Twenty-four (10%) of the 244 trials were identified as studying at least one of the two maintenance approaches (i.e., trial designs in Figure 1). Five (2%) trials studied radioimmunotherapy (Table 1).

A second search was done in NCI CTEP databases for active or completed maintenance clinical trials. Trials were counted if they tested drug-then-drug, drug-then-radiotherapy, or radiotherapy-then-drug sequential treatment for patients with any-type cancer and if they studied at least one outcome of clinical interest—toxicity, tumor response, progression-free survival, or overall survival. The search found no trial using the terms “switch maintenance” or “continuation maintenance”. The search identified 129 nonovarian trials; 27 (21%) trials fit the definition for a switch maintenance approach. No one solid tumor or hematological disease setting, nor choice of first-line or prior-line therapy, was preferred in these 27 trials. Of the 129 nonovarian trials, 21 (16%) were chemotherapy-then-radiotherapy trials and six (5%) were chemotherapy-then-immunotherapy trials. There were no (0%) radiotherapy-then-chemotherapy, immunotherapy-then-chemotherapy, immunotherapy-then-radiotherapy or radiotherapy-then-immunotherapy maintenance trials. One (1%) trial studied radioimmunotherapy (i.e., a radionuclide-antibody immunoconjugate) for nonovarian advanced-stage disease [15]. 

In our opinion, a better understanding of a radiopharmaceutical switch or continuation maintenance approach can be had from past choices of prior-line, maintenance, and next-line therapy. Given that maintenance radiopharmaceuticals are not considered second-line standard of care agents in most disease settings, we agree that trials should prespecify a second-line therapy (i.e., an alternative to the radiopharmaceutical) to be used at progression to ensure ease of trial interpretation. This recommendation has been stated before [7]. Four early-phase radioimmunotherapy trials that emphasize these points are summarized next. One phase III trial is not discussed within the scope of this manuscript because the randomly allocated standard treatment alone arm was not prespecified nor described, and thus this obscures trial interpretation and precludes review here [14].

The first radioimmunotherapy study involved switch maintenance. It administered first-line platinum-based chemotherapy followed later by maintenance radioimmunotherapy in the form of a lutetium-177 (^177^Lu)-CC49 antibody immunoconjugate [10]. ^177^Lu decays with a physical half-life of nearly 160 h. It emits therapeutic β particles (149 keV) that penetrate up to 600 μm in soft tissue, as well as γ rays (113, 208 keV) for diagnostic imaging [10]. CC49 is a murine monoclonal antibody that binds with high affinity to epithelial tumor surface-associated glycoprotein TAG-72.3 [16]. On trial, ^177^Lu-CC49 suspension was administered by gravity via an intraperitoneal fenestrated catheter, presuming that the suspension would coat all peritoneal surfaces after choreographed four-direction rolls repeated every 15 min over two hours. These 15 min rolls aided peritoneal surface coverage, as loculated pockets of peritoneal ascites blocked access to disease or concentrated ^177^Lu-CC49 in pools that would contribute to toxicity. This phase I trial with phase II expansion (1993–1995) enrolled 27 women with recurrent or refractory ovarian carcinoma after they had uniformly undergone six courses of cisplatin-paclitaxel chemotherapy (Table 1). Women were given a single intraperitoneal ^177^Lu-CC49 dosage (up to 45 mCi m^−2^). They mostly had grade 3 or higher (67%) stage IIIc abdominal disease (70%) with recurrent or refractory nodules measuring 1 cm or greater (48%). About two-thirds had no side effects, while 10 (37%) of 27 had arthralgia or myalgia (sometimes with fever). Transient neutropenia grade 2 or higher (11 (41%) of 27) was the most frequent hematological toxicity. Almost all (26 (96%) of 27) developed human anti-mouse antibodies (HAMA) about two weeks posttherapy, which are associated with reversible serum sickness. One (8%, treated at a 30 mCi m^−2^ dosage) of the 13 women who had a 4 cm mass achieved partial response without progression elsewhere that lasted for a 7-month period. Two (22%) of nine women whose nodules were measurable, but less than 1 cm, were progression-free for 4 and 5 months. Four (80%) of five women whose nodules were nonmeasurable were progression-free for six, 21, 32, and 35 months. Second-line therapy at progression was not prespecified, confounding interpretation of posttrial overall survival.

A second phase I trial (1996–2001) continued first-line paclitaxel in the maintenance course of the trial but in a new combination with the ^177^Lu-CC49 antibody immunoconjugate treatment (Table 1, [11]). This trial also added subcutaneous interferon (IFN) α2b injection to the regimen, as it had been shown both to increase TAG-72.3 antigen expression and improve radioimmunotherapy localization [17,18]. Paclitaxel was continued in the maintenance course for its radiosensitizing properties at the G2/M cell cycle phase [19]. As before, women changed position every 15 min over two hours to aid in ^177^Lu-CC49 peritoneal surface exposure. On trial, 44 women with recurrent or refractory TAG-72.3 positive ovarian carcinomas were given four subcutaneous IFN α2b (3 × 10^6^ units) injections on alternate days five days before, plus one intraperitoneal paclitaxel (0 to 100 mg m^−2^) instillation two days before, a single intraperitoneal ^177^Lu-CC49 dosage (32 to 45 mCi m^−2^). In these women, INF α2b administration increased the observed median whole body radioactive half-time (118 versus 93 h, *p* = 0.001) and the median whole-body radioactive dose (0.77 versus 0.68 cGy mCi^−1^, *p* = 0.02). The time to median peak ^177^Lu-CC49 plasma radioactivity (58 h) after its intraperitoneal instillation was not altered by IFN α2b injection (53 h, *p* = 0.47), or, IFN α2b injection plus paclitaxel intraperitoneal instillation (57 h, *p* = 0.73). Four (24%) of 17 patients with measurable disease achieved partial responses lasting three to nine months without progression. Four (24%) of 27 patients with nonmeasurable disease had progression-free intervals of 18, 21, 21, and 37 months. Again, second-line therapy at progression was not set at the beginning of the trial. This masks the impact of maintenance upon overall survival.

A third phase I trial (1999–2000) incorporated a continuation maintenance approach. It tested paclitaxel-^90^Yttrium (^90^Y)-CC49 antibody immunoconjugate treatment in 20 women with recurrent or refractory ovarian carcinoma (Table 1, [12]). Here, the ^90^Y radionuclide was substituted for ^177^Lu in the established regimen for its physical half-life of about 64 h, and, for its pure β particle emissions (935 keV) that more deeply penetrate soft tissue (up to 5000 μm) [12]. On trial, all 20 women underwent maximal safe cytoreductive abdominal surgery followed by six carboplatin–paclitaxel cycles in most (90%). Eleven (55%) had refractory disease at second-look laparotomy; nine (45%) had disease relapse at six months or greater. They most often had grade 3 or higher (60%) stage III abdominal disease (80%). They were given four dosages of subcutaneous IFN α2b (3 × 10^6^ units) on alternate days beginning five days before, and a single intraperitoneal instillation of paclitaxel (100 mg m^−2^) two days before, a single intraperitoneal ^90^Y-CC49 dosage (up to 24 mCi m^−2^). All 20 (100%) women developed hematological toxicity; reversible neutropenia grade 2 or higher (75%) was most frequent. Eight (40%) developed HAMA, peaking between four and six weeks posttherapy. Two (22% of nine) women with measurable disease achieved partial response, lasting two and four months. Among 11 women with nonmeasurable disease, four (36%) were progression-free for 15, 18, 19, and 23 months. As before, an impact of maintenance on overall survival was limited as posttrial next-line therapy was not prespecified.

Within the limits of interpretation, these three radioimmunotherapy trials provided proof-of-concept that an antibody-conjugated radiopharmaceutical targeting a tumor cell surface antigen delivers clinical benefit with a favorable safety profile in women with recurrent or refractory ovarian carcinoma. Uniform prior-line chemotherapy in these trials aided trial interpretation by standardizing the trial population under study. After these three trials were conducted, the supply of the murine CC49 antibody became depleted; other reagents were explored but not pursued. Second-line or next-line therapy was not stipulated on trial. Because the intensity of second-line or next-line treatment varied according to the agent(s) administered, true clinical benefit of the maintenance agent was undermined in these trials. HAMA and serum sickness were complications, but not dose-limiting barriers, to treatment on trial. Women were followed closely on these three trials. Such surveillance lessens problematic variances in clinical follow-up because trial variability in clinical visits raise observation bias or next-line treatment initiation bias.

A fourth radioimmunotherapy study (1995–1996) involved switch maintenance [13]. In this six-patient phase II trial of women with stage III epithelial ovarian carcinoma, debulking surgery preceded platinum-based chemotherapy and then was followed by second-look surgery and maintenance radioimmunotherapy as an iodine-131 (^131^I)-OC-125 antibody immunoconjugate. ^131^I decays with a physical half-life of 192 h and emits therapeutic β-particles (606 keV) that penetrate in soft tissue up to 800 μm. OC-125 is a murine monoclonal antibody that reacts with epithelial tumor surface-associated cancer antigen 125 (CA-125). On trial, the use of radioimmunotherapy was guided by the findings of second-look surgery—three (50%) had macroscopic (<5 mm) persistent disease and two (33%) had microscopic disease. One (17%) had macroscopic disease on surveillance imaging and did not undergo second-look surgery. Treatment involved Lugol’s solution (30 drops/day) given for three days before and six days after iodine treatment in order to block free ^131^I thyroid uptake. A two-liter ^99m^Tc colloid scintigraphic evaluation confirmed relatively uniform abdominopelvic distribution of the colloid before administering the therapeutic radioactive suspension. ^131^I-OC-125 suspension (120 mCi) was administered by gravity via an intraperitoneal fenestrated catheter, presuming that the suspension would distribute to all peritoneal surfaces after choreographed four-direction rolls repeated every 15 min for two hours. Two-thirds had protracted hematological toxicity (between 30 and 58 days after radioimmunotherapy [13]). All six (100%) women developed HAMA at two weeks posttherapy [13]. None achieved a response [13]. Third-line or greater therapy at progression was not prespecified on trial, and therefore, this aspect muddles interpretation of posttrial overall survival.

Targeted therapeutic radiopharmaceuticals hold great potential in the treatment of ovarian carcinoma because of the exact delivery of alpha or beta particle-emitting radionuclides to tumors [9]. These agents should have a high affinity for a targeted cancer antigen as well as have desirable metabolism and pharmacokinetic elimination factors that irradiate tumor and not normal tissue [9].

Thorium-227 (^227^Th) can be linked at ambient temperature to immunoconjugates like the anti-human epidermal growth factor receptor 2 (HER2) antibody trastuzumab bearing the chelator octadentate 3,2-hydroxypyridinone (3,2-HOPO) [20]. ^227^Th has a physical half-life of about 450 h, and, its α-particle emissions penetrate soft tissue up to 80 μm. However, patient selection for a maintenance trial using ^227^Th-HER2 trastuzumab is likely to be very narrow because only six percent of women with ovarian carcinoma harbor HER2+ overexpression [21,22]. Figure 1 provides one possible trial design. Here, eligibility-determining HER2-avid disease might be detected by zirconium-89 (^89^Zr)-labelled trastuzumab, as has been done already for breast and esophagogastric cancers [23,24]. The preclinical evaluation of the ^227^Th-HER2 trastuzumab conjugate demonstrated potent in vitro and in vivo activity in cellular and patient-derived xenograft (PDX) models of breast and gastric cancer [20]. Preclinical evaluation of ^227^Th-HER2 trastuzumab in HER2-positive cell-derived xenograft (CDX) ovarian carcinoma models also replicated this potency [25]. Other preclinical ovarian carcinoma model evaluations are instructive for ^89^Zr-trastuzumab diagnostic imaging [26] and radiopharmaceutical dosimetry [27]. For these reasons, NCI CTEP has added the ^227^Th-HER2 trastuzumab conjugate to its investigational agent portfolio to be used in next-generation radiopharmaceutical trials.

The ability to exchange radionuclides, like swapping lead-212 (^212^Pb) for ^227^Th on the trastuzumab antibody (using alternative linkers) [28,29], brings forth opportunities to study alternative toxicity profiles and clinical benefit. One phase I trial involved the agent ^212^Pb-S-2-(4-isothiocyanatobenzyl)-1,4,7,10-tetraaza-1,4,7,10-tetra(2-carbamoyl-methyl)cyclododecane trastuzumab (or, ^212^Pb-TCMC-trastuzumab) for women or men with recurrent or refractory HER2 1+ (10% tumor staining) or greater-expressing carcinomas disseminated in the abdominal cavity after at least one line of prior chemotherapy. ^212^Pb (half-life: ~11 h) decays to bismuth-212 (^212^Bi) by β-particle emission. ^212^Bi (half-life: ~1 h) decays to thallium-208 (^208^Tl, half-life: ~3 min) by α-particle emission (36%), or, to polonium-212 (^212^Po, half-life: 0.3 microseconds) by β-particle emission (64%). ^212^Po decays to ^208^Pb (stable) by α-particle emission. The two α-particles generated in this decay penetrate in soft tissue up to 50 μm. The investigators chose trastuzumab as the immunoconjugate, (i) as a humanized monoclonal antibody directed with high affinity against HER2 and (ii) because of its well-known safety profile and tolerable toxicity as monotherapy. On trial, a single ^212^Pb-TCMC-trastuzumab intraperitoneal injection (up to 0.74 mCi m^−2^ in 50 mL) was administered by fenestrated catheter less than four hours after a trastuzumab (4 mg kg^−1^) loading dosage. The loading dosage was given to saturate non-abdomen systemic body HER2 targets [28]. This phase I trial with expansion (2011–2014) enrolled 18 patients (16 women with recurrent or refractory ovarian carcinoma and two men with recurrent or refractory colon cancer). About three-fifths (62%) had no side effects, with six (38%) out of 16 describing grade 1 abdominal pain or tenderness and five (31%) with grade 2 or 3 infection. One (6%) instance of transient thrombocytopenia grade 1 was observed. None (0%) developed HAMA over a 6-week posttherapy observation period, and no one (0%) developed serum sickness. No (0%) partial or complete responses were documented; 44 percent (8 of 18) progressed within six weeks of therapy. Second-line therapy at disease progression was not prespecified on trial, again obscuring the interpretation of posttrial overall survival. A prospective trial engaging a radiopharmaceutical switch maintenance approach like in Figure 1 using a ^212^Pb-TCMC-trastuzumab immunoconjugate enrolling women with recurrent or refractory HER2-avid ovarian carcinoma might be of future interest. Identifying the triage radiotracer uptake agent would be needed.

## 3. Discussion

A maintenance approach intending clinical use of a radiopharmaceutical depends upon (i) the preferential expression of cancer-associated antigens by tumor cells as compared to normal cells, (ii) effective delivery and penetration of radionuclide emissions into the tumor, (iii) prolonged retention of the radionuclide at the tumor site, and (iv) radiation-induced nuclear DNA damage that kills cancer cells with minimal damage to normal cells. Unlike a setting for regulatory approval of a new treatment, where drug activity justifies drug market availability for patients with an indicated disease, a switch or continuation maintenance treatment setting tests with immediate rather than delayed exposure (i.e., treating at disease progression), is of clinical benefit [7]. In a maintenance trial, maximum benefit by using the putative maintenance agent early, as compared to later at the time of disease progression, is not fully vetted by a progression-free survival endpoint because unspecified next-line therapy variably impacts survival due to irregular biological or clinical activity [7]. In order to provide a sufficient level of evidence to inform clinical practice, trials evaluating switch or continuation maintenance should use, whenever possible, either overall survival as the endpoint, or, validated endpoints measuring quality of life [7]. To most accurately isolate the clinical impact of a maintenance approach, trials designs should incorporate randomization, uniform prior-line and prespecified next-line therapies, and similar between-arm follow-up schedules [7].

Maintenance approaches interrogate prolonged periods of ‘posttherapy’ agent exposure (i.e., after first-line treatment) using agents with clinical activity possibly established in more advanced-stage cancer settings. Bringing forth agents with already investigated clinical activity in advanced-stage cancer settings provides a more optimal assessment of clinical benefit in a radiopharmaceutical maintenance approach. For substantial gains to made, the radiopharmaceutical maintenance trial should be sized sufficiently to detect a meaningful improvement in overall survival over a reasonable study observation time period.

At the present, recurrent or refractory metastatic ovarian cancer patient survival is poor, and so, women with advanced-stage metastatic disease are subjects of interest for trials that examine radiopharmaceutical maintenance approaches. In other disease settings such as indolent neuroendocrine cancer, the overall survival endpoint might not be suitable for a reasonable trial observation timeframe due to the protracted survival of these patients. In this situation, endpoints that observe a reduction in the number of or the use of next-line therapies, or, a reduced number of medical interventions, draws attention to a meaningful therapeutic gain. So too, measured improvements in quality of life would assess an important clinical gain from a maintenance approach over a standard approach. As the fiscal debts and social distresses a patient incurs from the costs of cancer treatment rise [30], the clinical and social implications of a maintenance approach needs further study. This is because a longer treatment period from maintenance treatment would draw upon more public healthcare resources [30]. Therefore, in our opinion, it is vital for radiopharmaceutical maintenance approaches to evaluate clinical benefit of maintenance through randomized clinical trials, with endpoints measuring overall survival or quality of life.

## 4. Materials and Methods

We searched academic scholarship for studies invoking a switch or continuation maintenance anticancer treatment strategy from among peer-reviewed articles in PubMed (www.pubmed.gov) available between January 1966 and June 2020 or in Google Scholar (scholar.google.com) available between January 1995 and June 2020. Search terms included “clinical trial”, “switch maintenance”, “cisplatin”, “carboplatin”, “poly(ADP-ribose) polymerase (PARP)”, “immunotherapy”, “radiotherapy”, “radioimmunotherapy”, or “radiopharmaceutical”, and “ovarian carcinoma” or “ovarian carcinoma”. Articles available in the English language were read for primary research hypotheses, safety or efficacy findings, limitations, and data explanations. Summaries and proportions are provided.

A search of the NCI CTEP Integrated Platform for Agents and Diseases (IPAD) database (version 6.16.0, Rockville, MD, USA) was performed to count the number of switch or continuation maintenance trials that had been activated or completed between April 1974 and June 2020. Clinical trials were sorted using search terms of “chemotherapy”, “immunotherapy”, “radiotherapy”, phase “0, I, I/II, II, II/III, or III” and “active, complete, closed to accrual, or closed to accrual & treatment”. Duplicates from the academic scholarship search were removed. Summaries and proportions are used.

## 5. Conclusions

In conclusion, radioimmunotherapy trials establish a foundation upon which next generation therapeutic radiopharmaceutical maintenance trials can be designed. In our opinion, a radiopharmaceutical maintenance therapy question should test an immediate exposure treatment approach against a treatment at progression approach that involves a targeted therapeutic agent. Use of HER2-targeting radiopharmaceutical immunoconjugates was introduced as an intriguing approach to maintenance treatment among women with recurrent or refractory ovarian carcinoma. Our review of available scholarship suggests that radiopharmaceutical maintenance trial designs should incorporate either overall survival or quality of life as the primary trial endpoint. In our opinion, radiopharmaceutical switch or continuation maintenance for women with recurrent or refractory ovarian carcinoma should be considered in prospective clinical trials.

## Figures and Tables

**Table 1:** Switch or continuation maintenance in ovarian carcinoma and selected trial characteristics.

Disease Setting	N	Agents	Treatment Regimen			Platinum-Sensitive Patients		Ref.
			Dose	Day	Cycle(s)	(%) <6 mo.	(%) ≥6 mo.	
recurrent or refractory	27	^177^lutetium-CC49 antibody	45 mCi m^−2^ (1665 MBq)	1	1	3 (11)	24 (89)	[10]
recurrent or refractory	44	^177^lutetium-CC49 antibody paclitaxel	40 mCi m^−2^ (1480 MBq)100 mg m^−2^	1 −2	1	43 (98)	1 (2)	[11]
recurrent or refractory	20	^90^yttrium-CC49 antibody paclitaxel	24 mCi m^−2^ (888 MBq)100 mg m^−2^	1 −2	1	11 (55)	9 (45)	[12]
post-second look surgery	6	^131^iodine- OC-125 antibody	120 mCi (4440 MBq)	1	1	6 (100)	0 (0)	[13]
post-second look surgery	447	^90^yttrium-muHMFG1 antibody	18 mCi m^−2^ (666 MBq)	1	1	99 (44)	143 (56)	[14]

Abbreviations: AUC = area under the curve; m = meter; mCi = millicurie; mg = milligram; mo. = month; *N* = number; Ref = reference.
